# ZNF445 is a primary regulator of genomic imprinting

**DOI:** 10.1101/gad.320069.118

**Published:** 2019-01-01

**Authors:** Nozomi Takahashi, Andrea Coluccio, Christian W. Thorball, Evarist Planet, Hui Shi, Sandra Offner, Priscilla Turelli, Michael Imbeault, Anne C. Ferguson-Smith, Didier Trono

**Affiliations:** 1 Department of Genetics, University of Cambridge, Cambridge CB2 3EH, United Kingdom;; 2 School of Life Sciences, Ecole Polytechnique Federale de Lausanne (EPFL), Lausanne 1015, Switzerland

**Keywords:** KRAB zinc finger proteins, ZFP445, ZFP57, genomic imprint maintenance, resistance to epigenetic reprogramming

## Abstract

Genomic imprinting is an epigenetic process regulated by germline-derived DNA methylation, causing parental origin-specific monoallelic gene expression. Zinc finger protein 57 (ZFP57) is critical for maintenance of this epigenetic memory during post-fertilization reprogramming, yet incomplete penetrance of *ZFP57* mutations in humans and mice suggests additional effectors. We reveal that ZNF445/ZFP445, which we trace to the origins of imprinting, binds imprinting control regions (ICRs) in mice and humans. In mice, ZFP445 and ZFP57 act together, maintaining all but one ICR in vivo, whereas earlier embryonic expression of ZNF445 and its intolerance to loss-of-function mutations indicate greater importance in the maintenance of human imprints.

## Abstract (precis)

In this study, Takahashi et al. show that ZNF445/ZFP445 binds imprinting control regions (ICRs) in mice and humans. In mice, ZFP445 and ZFP57 act together, maintaining all but one ICR in vivo, whereas earlier embryonic expression of ZNF445 and its intolerance to loss of function mutations indicate greater importance in the maintenance of human imprints.

The Krüppel-associated box (KRAB)-containing zinc finger protein (KZFP) represents one of the fastest evolving gene families in the human genome. In general, they function to recruit repressive epigenetic states to transposable elements in a species-specific manner (Imbeault et al. 2017), but evidence for a role for these proteins in the regulation of unique genomic regions has emerged recently. Genomic imprinting causes the parental origin-restricted expression of ∼100 genes in humans and mice due to germline-derived differential DNA methylation at imprinting control regions (ICRs) (Ferguson-Smith 2011). In particular, the KZFP ZFP57 protects these methylation imprints from genome-wide erasure during the preimplantation period through its methylation-dependent recognition of the TGC^m^CGC sequence present in all murine ICRs and most human putative ICRs (Supplemental Table S1), where it recruits DNA methyltransferases, KAP1 (KRAB-associated protein 1, also called TRIM28), and histone methyltransferases (Strogantsev et al. 2015). ZFP57 is essential for imprinting maintenance at all ICRs in cultured murine embryonic stem cells (mESCs) (Riso et al. 2016), but only a subset of imprints is lost in maternal–zygotic *Zfp57* mutant mice (Li et al. 2008; Takahashi et al. 2016). In humans, the phenotype associated with mutations in *ZFP57* is even milder, since, in homozygous recessive patients with transient neonatal diabetes mellitus (TNDM), a form of multilocus imprinting disturbance (MLID), *ZFP57* influences only a minority of imprinted differentially methylated regions (DMRs) (Boonen et al. 2013; Court et al. 2013; Bak et al. 2016). Moreover, the absence of ZFP57 in human oocytes suggests a less prominent role for this protein compared with its murine ortholog and prompted us to identify another factor that could complement its function (Okae et al. 2014). We hypothesized that this additional effector was another member of the KZFP family and identified ZNF445/ZFP445 as the missing regulator required for the maintenance of post-fertilization germline methylation imprints.

## Results and Discussion

### ZNF445/ZFP445 binds at ICRs in human ESCs (hESCs) and mESCs

**Figure 1.**
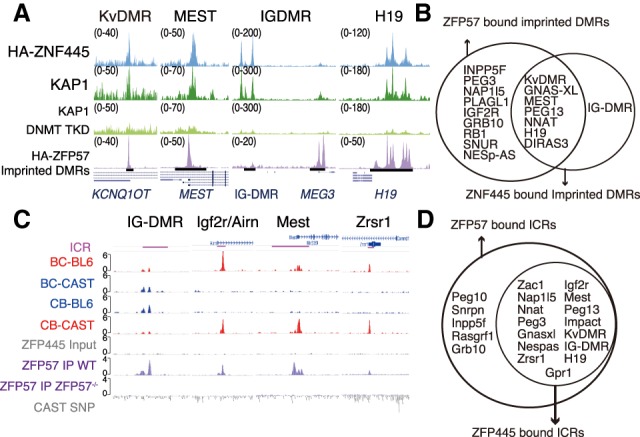
ZNF445/ZFP445 binds ICRs in hESCs and mESCs. (*A*) Human: screenshot of ChIP-seq data at genomic loci corresponding to the indicated imprinted DMRs (black bars), illustrating enrichment for KAP1 in untreated hESCs or cells with knockdown for the three DNA methyltransferases (DNMT TKD) or for ZNF445 and ZFP57 in cells overexpressing HA-tagged versions of either protein. (*B*) Venn diagram of imprinted DMRs bound by ZFP57 and/or ZNF445 in hESCs. (*C*) Mouse: screenshot of ChIP-seq for ZFP445 and ZFP57 in mESCs. For ZFP445, mESCs obtained from C57BL/6J × CAST/Ei and reciprocal CAST/Ei × C57BL/6J crosses were used. Single-nucleotide polymorphisms (SNPs) between the two mouse strains were used to assign the reads to the parental alleles. (Red) Maternal; (blue) paternal. (*D*) Venn diagram of ICRs bound by ZFP57 and/or ZFP445 in mESCs.

We first screened our database of human KZFPs genomic binding sites in HEK293T cells (Imbeault et al. 2017) for those interacting with germline DMRs (Okae et al. 2014). ZFP57 was enriched at 17 of 31 of these DMRs, and we identified ZNF445, another KZFP, at 12 (Supplemental Fig. S1A; Supplemental Table S1). We confirmed these data through chromatin immunoprecipitation (ChIP) combined with high-throughput sequencing (ChIP-seq) of hESCs overexpressing either protein (Supplemental Fig. S1B), finding ZFP57 associated with 17 and ZNF445 associated with eight germline DMRs (Fig. 1A,B; Supplemental Table S1). In contrast to most KZFPs, ZNF445 was not significantly associated with transposable elements (Supplemental Fig. S1C). Interestingly, the DMRs most affected in *ZFP57*-mutated human subjects (*PEG3*, *PLAGL1*, *INPPF5*, *NAP1l5*, and *GRB10*) (Boonen et al. 2013; Court et al. 2013; Bak et al. 2016) were not bound by ZNF445 in hESCs (Fig. 1B; Supplemental Table S1). The two KZFPs generated overlapping binding profiles at maternal DMRs (such as *Kv*DMR and *MEST*), whereas, on paternal DMRs, they occupied distinct genomic positions, especially in the case of the *DLK1-DIO3* imprinted locus, where ZNF445 was bound to the human germline-inherited DMR IG-DMR, whereas ZFP57 was found at the somatic DMR at the *MEG3* promoter (Fig. 1A). *ZFP57* expression is barely detectable in hESCs (Supplemental Fig. S1D), and, accordingly, the recruitment of KAP1 to the DMRs matched that of ZNF445 rather than ZFP57 in these cells (Fig. 1A). Furthermore, these ZNF445-matching KAP1 peaks were lost upon knockdown of the three DNA methyltransferases (*DNMT1*, *DNMT3A*, and *DNMT3B*) in hESCs, suggesting that the binding of the KZFP responsible for its genomic recruitment is dependent on DNA methylation (Castro-Diaz et al. 2014). Binding of endogenous ZNF445 to these DMRs was confirmed by ChIP-qPCR (ChIP combined with quantitative PCR), excluding potential artifacts linked to overexpression (Supplemental Fig. S1E).

To determine whether the murine ortholog ZFP445 also bound at imprinted loci, we performed ChIP-seq of its HA-tagged form overexpressed in hybrid mESCs derived from reciprocal crosses between the *Mus musculus domesticus* (C57BL/6J) and *M. musculus Castaneus* (CAST/Ei) strains (Supplemental Fig. S2A), allowing the parental origin of alleles to be determined. We compared our results with ZFP57-binding data from the same reciprocal hybrid cells (Strogantsev et al. 2015). We found enrichment for both ZFP57 and ZFP445 at 15 ICRs, with another five ICRs bound by ZFP57 alone (Fig. 1C,D; Supplemental Fig. S2B; Strogantsev et al. 2015). In all cases, like ZFP57, ZFP445 was associated with the strain harboring the methylated allele, and, also consistent with ZFP57 data, the binding of ZFP445 to ICRs was lost in cells deprived of DNA methylation by triple knockout of the DNA methyltransferase-coding genes *Dnmt1*, *Dnmt3a*, and *Dnmt3b* (Supplemental Fig. S2C; Tsumura et al. 2006), indicating methylation-sensitive binding properties.

### ZFP57 and ZFP445 together maintain mouse imprints in vivo

**Figure 2.**
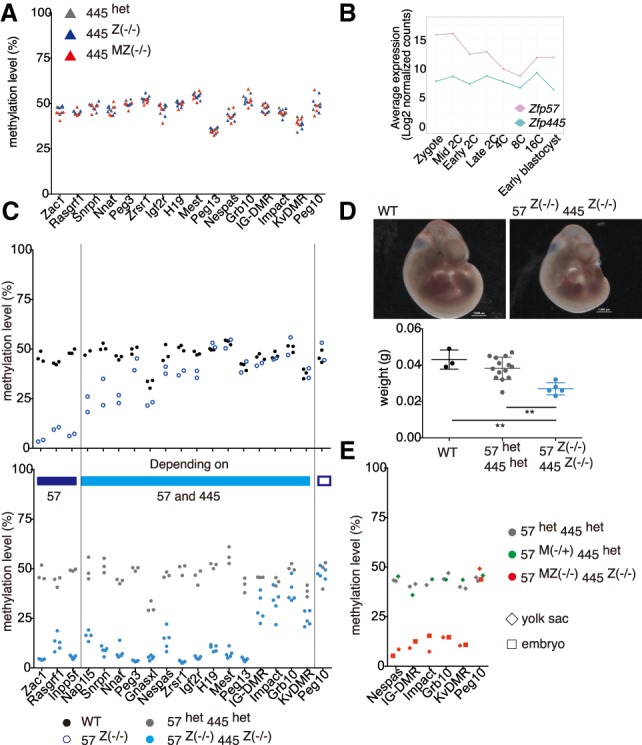
ZFP57 and ZFP445 together are required to maintain imprint methylation in vivo. (*A*) Methylation levels at ICRs in the brains of E12.5 embryos in the indicated genetic mutants. *n* = 4. Each dot represents an average of methylation at ICR CpG sites using quantitative pyrosequencing. (*B*) Expression of *Zfp57* and *Zfp445* in murine early development (Deng et al. 2014). (*C*) Methylation levels measured by pyrosequencing in embryonic brains of the indicated genetic mutants at E11.5. Each dot represents average methylation levels of analyzed CpG sites. Wild-type (*n* = 2), *Zfp57* ^Z(−/−)^ (*n* = 2), *Zfp57*^het^/*Zfp445*^het^ (*n* = 3), and *Zfp57*^Z(−/−)^/*Zfp445*^Z(−/−)^ (*n* = 5) embryos were obtained from six litters by crossing female and male double-heterozygous mutants. (*D*) Images of representative wild-type and *Zfp57*^Z(−/−)^/*Zfp445*^Z(−/−)^ E11.5 embryos and weights of E11.5 embryos in wild type (*n* = 3), *Zfp57*^het^/*Zfp445*^het^ (*n* = 14), *Zfp57*^Z(−/−)^ /*Zfp445*^Z(−/−)^ (*n* = 5) from six litters. (**) *P* < 0.01, one-way ANOVA. (*E*) Methylation levels measured by pyrosequencing in yolk sacs and embryos of the indicated genetic mutants. *Zfp57*^het^/*Zfp445*^het^ (*n* = 2) mutants were obtained from one litter by crossing female and male double heterozygotes, and *Zfp57*^MZ(−/−)^/*Zfp445*^Z(−/−)^ (*n* = 1) and *Zfp57*^M(−/+)^/*Zfp445*^het^ (*n* = 1) mutants were obtained by crossing female *Zfp57*^Z(−/−)^/*Zfp445*^het^ and male *Zfp57*^het^/*Zfp445*^Z(−/−)^ mutants. Images of those embryos are shown in Supplemental Figure S7.

To ask whether *Zfp445* might contribute to imprint regulation in vivo, we examined mice carrying either zygotic or maternal–zygotic *Zfp445* deletions (Supplemental Fig. S3A). About one-third of the *Zfp445* zygotic mutants survived to adulthood, a much milder phenotype than observed with zygotic *Zfp57* mutations (Supplemental Table S2A). Furthermore, *Zfp445* mutants did not exhibit any loss of methylation imprints at ICRs analyzed in the brain and liver at embryonic day 12.5 (E12.5), including those bound by ZFP445 in mESCs (Fig. 2A; Supplemental Fig. S3B). Consistent with no change in the methylation imprints, expression of imprinted genes was unperturbed in *Zfp445* mutants (Supplemental Fig. S3C). We conclude that the absence of ZFP445 alone has no impact on imprinting during mouse early development, perhaps due to compensation by high levels of ZFP57 (Fig. 2B).

Maternal–zygotic deletion of murine *Zfp57* alone results in complete, major, or partial loss of methylation at multiple ICRs except *H19*, *Kv*DMR, and *Peg10* (Supplemental Fig. S4A), while zygotic depletion induced total loss of DNA methylation at only three ICRs (*Inpp5f*, *Zac1*, and *Rasgrf1*) and partial loss at another eight ICRs. To determine whether compensation by *Zfp445* might explain the persistence of some imprinting in these *Zfp57* mutants, we generated *Zfp445*–*Zfp57* double-mutant mice (Supplemental Fig. S5; Supplemental Table S2B). Homozygous zygotic mutations for both genes caused embryonic lethality and showed no gross morphological abnormalities but significant reduction in size and weight at E11.5 (Fig. 2D), a phenotype more pronounced than observed in *Zfp57* mutant mice (Supplemental Table S2C). Correspondingly, *Zfp57/Zfp445* zygotic inactivation resulted in more severe loss of imprinting at 15 ICRs than solo *Zfp57* zygotic mutation (Fig. 2C; Supplemental Fig. S4B). In addition to the three ICRs that were devoid of methylation in *Zfp57* zygotic mutants, 11 more completely lost their imprints in the double mutants (Fig. 2C; Supplemental Fig. S4B). Thus, zygotic ZFP57 and ZFP445 are enough to protect methylation at 14 out of the 19 ICRs analyzed. Interestingly, *Peg10* was the only ICR found to be entirely unaffected in embryos homozygous for both mutations.

Partial loss of methylation at four ICRs (*IG*-DMR, *Impact*, *Grb10*, and *Kv*DMR) in the double-zygotic mutants suggested that maternal ZFP57 might attenuate the phenotype of the double-zygotic mutants, and thus we assessed the impact of *Zfp445* inactivation in a maternal/zygotic *Zfp57* knockout mouse (Supplemental Fig. S5). These *Zfp57*^*MZ(−/−)*^/*Zfp445*^*het*^ mutants exhibited a more severe imprinting defect at nine ICRs compared with maternal–zygotic *Zfp57* mutants and at two ICRs (*IG*-DMR and *Impact*) compared with double-zygotic *Zfp57/Zfp445* mutants (Supplemental Fig. S4A,B). Interestingly, only *H19*, which is one of the most ancient ICRs conserved in marsupials (Smits et al. 2008), was markedly less affected in *Zfp57*^*MZ(−/−)*^/*Zfp445*^*het*^ than the double-zygotic mutant, indicating that *H19* has a stronger dependency on ZFP445 compared with other ICRs. Finally, we were able to generate a *Zfp57*^*MZ(−/−)*^/*Zfp445*^*Z(−/−)*^ embryo despite the high level of embryonic lethality of the required *Zfp57*^*Z(−/−)*^/*Zfp445*^*het*^ mutant mother (Supplemental Fig. S5). Indeed, only two surviving female *Zfp57*^*Z(−/−)*^/ *Zfp445*^*het*^ mice could be obtained from 134 litters (Supplemental Table S2C–G). The first female had five conceptuses at day 11.5 of gestation, with one being the sought-after *Zfp57*^*MZ(−/−)*^/*Zfp445*^*Z(−/−)*^ mutant; this appeared to have stopped developing around E10 (Supplemental Fig. S7). The second female had nine conceptuses at day 10.5 gestation, and none of them was a *Zfp57*^*MZ(−/−)*^/*Zfp445*^*Z(−/−)*^ mutant (Supplemental Table S2H). We analyzed methylation of the *Zfp57*^*MZ(−/−)*^/ *Zfp445*^*Z(−/−)*^ mutant using both yolk sac and embryo and confirmed that methylation was maintained at *Peg10* but was negligible or completely lost at all of the ICRs previously shown to be resistant to zygotic loss of ZFP57 with ZFP455 (Fig. 2E). Our results demonstrate that ZFP57 and zygotic ZFP445 cooperate to protect all but one ICR (*Peg10*) during mouse embryonic development.

### ZNF445 controls imprints in hESCs

**Figure 3.**
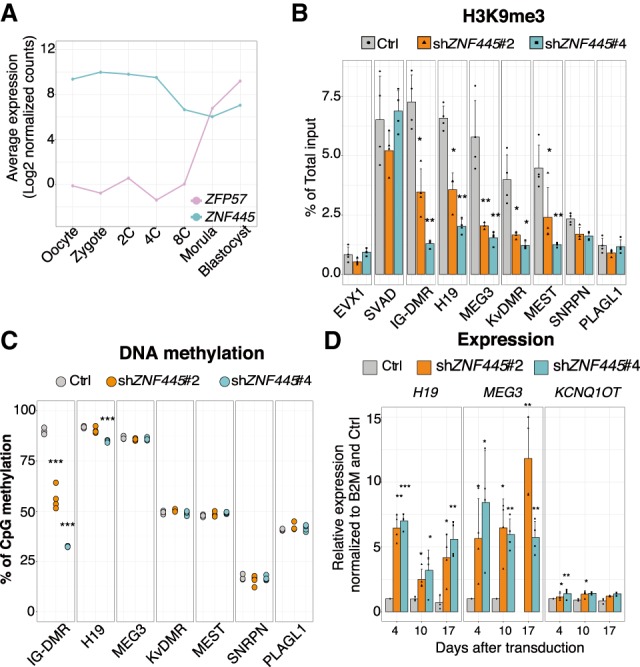
ZNF445 recruits H3K9me3 and regulates expression of imprinted genes in hESCs. (*A*) Expression of *ZFP57* and *ZNF445* in human early development (Yan et al. 2013). (*B*) H3K9me3 enrichment at the indicated genomic loci found by ChIP-qPCR in wild-type and *ZNF445* knockdown hESCs (using two different shRNAs). The bars represent the mean + SD, and single values are plotted for each replicate. (*) *P* < 0.05; (**) *P* < 0.01, Student's *t*-test. *n* = 4. (*C*) Dot plot representing the percentage of methylation measured by pyrosequencing at the indicated imprinted DMRs in control and *ZNF445* knockdown hESCs. Each point represents a different replicate. (***) *P* < 0.001, Student's *t*-test. *n* = 4. (*D*) Relative expression of imprinted genes as measured by RT-qPCR in *ZNF445* knockdown and control hESCs. Data were normalized to the *B2M* housekeeping gene. (*) *P* < 0.05, Student's *t*-test, *n* = 4.

In contrast to mice, human *ZFP57* transcripts are undetectable in the oocyte and during the earliest stages of embryonic development, increasing only after zygotic genome activation (Fig. 3A). This leaves a time window of several cell divisions, during which ZNF445 is potentially acting alone to protect human imprints from erasure. ESCs are the closest available in vitro model of early human embryogenesis even though they are prone to aberrant methylation imprints (Rugg-Gunn et al. 2007). We thus used hESCs to assess the ability of ZNF445 to maintain DNA methylation imprints, recruit KAP1 and histone 3 Lys9 methylation (H3K9me3) at imprinted DMRs, and influence the expression of imprinted genes. Only four germline DMRs (IG-DMR, *H19*, *Kv*DMR, and *MEST*) showed binding of both ZNF445 and KAP1 in hESCs (Supplemental Table S1) and thus are more likely to remain controlled by the two proteins in this cellular model. We knocked down *ZNF445* expression in hESCs by RNAi using two different shRNAs (Supplemental Fig. S8A,B). This resulted in loss of KAP1 binding and H3K9me3 enrichment at ZNF445/KAP1-bound imprinted DMRs (Fig. 3B; Supplemental Fig. S8C) and a drop in DNA methylation and up-regulation at the imprinted genes *MEG3* and *H19* (and, to a lesser extent, *KCNQ1OT*) (Fig. 3C,D; Supplemental Fig. S8B). Deregulation of *MEG3* and *H19* imprinted genes and loss of H3K9me3 were fully rescued by overexpressing a shRNA-resistant form of *ZNF445* and could be only partially compensated for by forced expression of ZFP57 (Supplemental Fig. S9). Interestingly the IG-DMR, which is the ICR most consistently affected upon *ZNF445* knockdown even when ZFP57 is overexpressed (Supplemental Fig. S9), is not perturbed in human patients with *ZFP57* mutations, correlating with the presence of only one ZFP57-binding motif in its sequence (Supplemental Fig. S4B). These data confirm the functional ability of ZNF445 to (1) bind human ICRs, (2) maintain DNA methylation at the IG-DMR and H19 DMR, (3) recruit KAP1 and trigger deposition of H3K9me3, and (4) regulate expression of a subset of imprinted genes in hESCs. Contrary to the mouse model, removal of ZNF445 alone was sufficient to affect the epigenetic status of ICRs and the expression of imprinted genes.

### ZNF445 evolved as the primary protector of imprints

**Figure 4.**
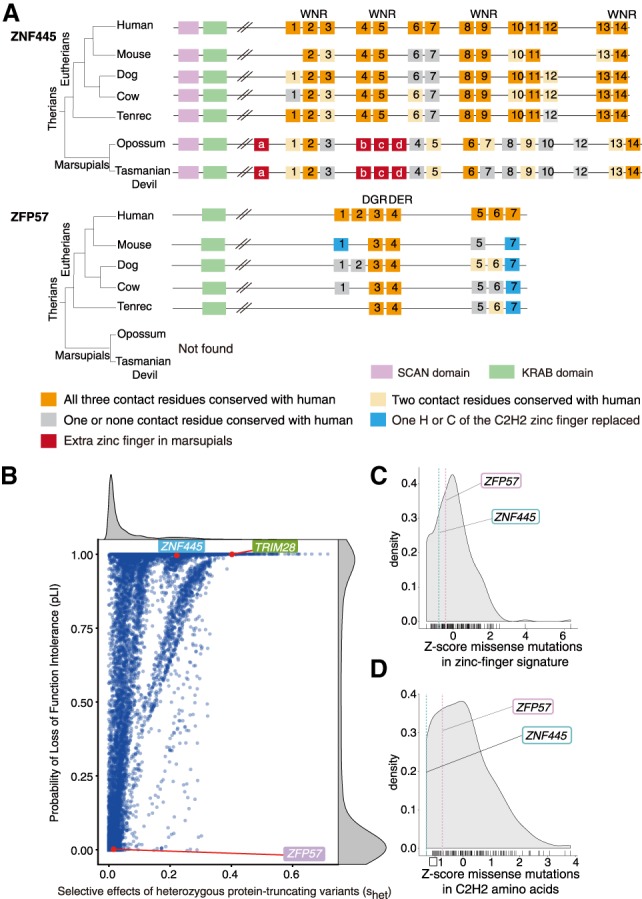
ZNF445 is highly conserved in therians and in the human population. (*A*) Schematic representation of the comparison between the DNA-binding signature of the zinc finger domains of ZNF445 and ZFP57 in various species. (*B*) Correlation between pLI (probability of loss-of-function intolerance) and S_het_ score for all of the genes in the human genome. (*C*,*D*) *Z*-scores for the occurrence of missense variants at the positions predicted to dictate the DNA-binding specificity of the zinc finger motifs (*B*) or on the structural C_2_H_2_ residues (*C*) for all of the KZFPs. Lower values indicate increased constraint.

Given the different roles played by *ZNF445* and *ZFP57* in mice and humans, we sought to retrace their evolutionary history. We used a previously described approach based on homologies in the so-called “zinc fingerprint” of KZFPs, predictive of their DNA-binding specificity (Liu et al. 2014). In two marsupials (opossum and Tasmanian devil), we identified putative *ZNF445* orthologs displaying arrays of zinc fingers reminiscent of their human and mouse counterparts. Interestingly, the marsupial genome does not harbor a sequence predicted to encode a product with the unique DGR–DER zinc finger pair characteristic of all *ZFP57* orthologs (Fig. 4A; Imbeault et al. 2017). Common properties of the putative *ZNF445* orthologs include the highly conserved WNR DNA-binding signature and their location in genomic neighborhoods of syntenic homology next to *ZKSCAN7* orthologs (Fig. 4A; Supplemental Fig. S10). Marsupials have been shown to have imprinted expression of a subpopulation of the genes that are imprinted in eutherians, including *H19-Igf2* and *Peg10* (Suzuki et al. 2007; Smits et al. 2008), neither of which is regulated by ZFP57 alone (Supplemental Fig. S4A). Intriguingly, Peg10 is a neogene derived from a Ty-3 Gypsy retrotransposon of the Sushi-ichi class (Youngson et al. 2005), and, as the wider repertoire of KZFPs evolved to target repressive epigenetic states to transposable elements (Imbeault et al. 2017), it is possible that maintenance of its imprint is safeguarded independently by yet another KZFP. *ZNF445* thus appears to have preceded *ZFP57* in mammalian evolution, likely emerging just before the separation between Eutheria and Metatheria, since no *ZNF445* ortholog is detected in egg-laying monotremes where imprints have not been found.

To further probe a potential role for *ZNF445* in human early development, we analyzed its genetic variation in the general population using exome and whole-genome sequencing data from 123,136 and 15,496 individuals, respectively, available through the gnomAD database (Fig. 4B; Lek et al. 2016). We found the probability of loss-of-function intolerance (pLI) (Lek et al. 2016) to have the same maximal value of 1.0 for *ZNF445* and *KAP1*, indicating that even heterozygous inactivating mutations in these genes confer haploinsufficiency, consistent with the *Zfp445* dosage sensitivity observed in *Zfp57*^*Z(−/−)*^ and *Zfp57*^*MZ(−/−)*^ mice (Supplemental Figs. S4, S6). The S_het_ score (Cassa et al. 2017), which estimates the selection against heterozygous loss of function, further confirmed very strong selective pressures on *ZNF445* and *KAP1*, with a lower mutational tolerance for *ZNF445* than for *ZFP57*, although both genes have a minimal rate of variation and display strong conservation at sequences coding for amino acids important for the structure or the DNA-binding specificity of their zinc finger arrays (Fig. 4C,D; Supplemental Fig. S11). It suggests that mutations in *ZNF445*, albeit rare in the human population, might constitute the basis for severe multilocus imprinting disorders and for unexplained cases of infertility and miscarriage.

In conclusion, our study reveals an important role for the evolutionarily conserved ZNF445/ZFP445 in the regulation of imprinting. In mice, ZFP57 plays the predominant role in imprinting maintenance, while, in its absence, ZFP445 is required for preserving methylation at a subset of ICRs. In humans, the lack of maternal ZFP57 and the mild effects of *ZFP57* mutations on imprints argue for a less prominent role of this protein in imprinting maintenance. The expression profile of *ZNF445*, its intolerance to loss-of-function mutations, and the ability of its product to bind and instate heterochromatin at ICRs strongly suggest that ZNF445 is a major factor in human early embryonic imprinting maintenance. Our evolutionary analysis further suggests that *ZNF445* might have been the first KZFP that evolved to control imprinting. In humans, it has retained a central role, while *ZFP57*, which likely emerged later, became functionally more prominent in rodents. Thus, our study uncovers the parallel evolution of two KZFPs involved in the regulation of an essential mammalian epigenetic process and portrays how different evolutionary lineages have balanced the relative functional impact of these two factors and differentially modulated their transcriptional regulation during early development.

## Materials and methods

### Mice

All mouse work was conducted under a project license from the UK Government Home Office. ZFP445 mutant mice on C57BL/6N [Zfp445^tm1a(EUCOMM)Hmgu^] were obtained from the International Mouse Phenotyping Consortium. ZFP57 mutants (Takahashi et al. 2016) were maintained on C57BL/6. For generating maternal–zygotic mutants, animals were backcrossed to 129aa for >12 generations to obtain zygotic mutant adults that are inviable on C57BL/6. Mice were housed in a temperature- and humidity-controlled room under 12-h light/12-h dark cycles. All mice were ear-notched and genotyped by PCR using PCRBIO Rapid Extract lysis kit (PCR Biosystems) before postnatal day 10. Fetuses at E11.5 and E12.5 were collected, weighed, and photographed, and then tissues were dissected in PBS.

### DNA methylation analysis

The procedure for DNA methylation analysis was described previously (Strogantsev et al. 2015). All primers are listed in Supplemental Table S3.

### Cell culture and transduction

mESCs were cultured in 2i + LIF medium, and the hESC line (WA01, WiCell) was cultured in mTeSR1 medium (Stem Cell Technologies) on hES-qualified Matrigel (BD Biosciences) and in the presence of ROCK inhibitor (Y-27632). Cells deficient for *Dnmt1*, *Dnmt3a*, and *Dnmt3b* (Tsumura et al. 2006) were obtained from Professor Masaki Okano. pLKO.puro shRNA vectors were used for *ZNF445* knockdown. The shRNAs for *ZNF445* were obtained from the RNAi Consortium. All shRNAs sequences are listed in Supplemental Table S3. GFP, *ZNF445*, and *ZFP57* cDNAs were cloned in the pAIB HIV-1-based transfer vector by using an In-Fusion HD cloning kit (Clontech). The *Zfp57-*expressing vector was obtained from previous work (Gubelmann et al. 2013). *Zfp445* cDNA was codon-optimized, synthetized into pENTR vectors, and further transferred via gateway cloning into a puromycin-selectable lentivector under a tetracyclin-inducible TRE promoter to obtain HA-tagged proteins (pSIN-TRE-R1R2-3xHA).

### ChIP-PCR and ChIP-seq

Cells were harvested and fixed with 1% formaldehyde and quenched with 250 mM TrisHCl. Isolated chromatin was sonicated (Covaris), and immunoprecipitations were performed with chromatin from 1 × 10^7^ cells with Dynabeads (Thermo Fisher) in immunoprecipitation buffer (16.25 mM Tris at pH 8.1, 137.5 mM NaCl, 1 mM EDTA, 0.5 mM EGTA, 1.25% Triton X-100, protease inhibitors) overnight. Antibodies used were anti-HA.11 (Covance), H3K9me3 (Diagenode, C15410056), KAP1 (Millipore, MAB3662), and ZNF445 (Thermo Fisher, PA5-52322). ChIP samples were used for SYBR Green qPCR (Applied Biosystems) or library preparation for sequencing. All primers sequences are listed in Supplemental Table S3. Libraries of immunoprecipitated chromatin and total input control from ChIP were generated with paired-end adaptors as described previously (Ecco et al. 2016). Sequencing was performed on an Illumina NextSeq 500 (Illumina), with each library sequenced in 75-base-pair (bp) reads paired-end run or 100-bp single-end run.

### RT-qPCR

RT-qPCR was described previously (Coluccio et al. 2018). All primers are listed in Supplemental Table S3.

### Bioinformatic and statistical methods

R version 3.1.2 or Graphpad Prism version 4.0 was used for statistical analyses.

ChIP-seq analyses in human cells: For previously published data sets, raw data are available at GSE57989 (KAP1 hESCs) and GSE78099 (KRAB-ZFPs in HEK293Ts). Reads were mapped to human assembly hg19 using Bowtie2 short read aligner (Langmead and Salzberg 2012) using the --sensitive-local mode. The peaks were called using the MACS program version 1.4.2.1 (Zhang et al. 2008) with the total input chromatin coverage as control. For MACS, we used the default software parameters and selected a MACS score >50.

For ChIP-seq analyses in mESCs, reads after quality control were aligned to the mouse reference genome (mm10) with Bowtie2 (version 2.3.3, --end-to-end enabled). Potential PCR duplicates were removed with Picard tools (“MarkDuplicates” function). Peak calling was performed with MACS2 (version 2.1.0) (Feng et al. 2012) with the broad peak option using only uniquely aligned reads. ZFP445 peaks were normalized to the corresponding input control. Parental origin-specific binding was characterized by taking advantage of ∼21 million single-nucleotide polymorphisms (SNPs) that are present between the BL6 and CAST genome. Reads were mapped to merged BL6/CAST genomes, and subsequent deconvolution was undertaken using a custom Perl script. Visualization tracks were generated with BEDtools2 genomecov (version 2.27.0, -pc -bga -scale) with the scaling factor being per million (10^6^) the number of aligned reads. Visualization of ChIP-seq data was performed with the Washington University Epigenome Browser (Zhou et al. 2015).

Enrichment analysis on genomic features was performed with BEDtools software to generate intersection, shuffle tracks, and calculate *P*-values from Fisher exact test.

For RNA sequencing, human and murine early embryonic development data were taken from GSE36552 and GSE45719, respectively, and reanalyzed as described previously (De Iaco et al. 2017).

For human genetic analyses, genomic and variant data were obtained from 123,136 unrelated human exomes and 15,496 whole human genomes from gnomAD (release 170228) (Lek et al. 2016). Only variants annotated as passing quality thresholds with “PASS” were retained for the analyses. Gene and transcript data were obtained from Ensembl version 75 (hg19), with all analyses being performed on the canonical transcript, as defined by Ensembl. For all analyses, only single-nucleotide variants (SNVs) were included.

The pLI scores were obtained from the first published gnomAD data set containing 60,706 exomes, with the absence of additional or novel loss-of-function variants that could influence the original pLI scores confirmed using the latest release with 123,136 exomes. The S_het_ scores were obtained from a previous study (Cassa et al. 2017). The pLI and S_het_ scores differed in the statistical models used (posterior probabilities vs. Bayesian estimation). However, both scores were calculated on the basis of the observed number of protein-truncating variants in the same 60,706 exomes from gnomAD, although S_het_ also excluded frameshift variants from the statistical model.

The C_2_H_2_ zinc finger domains were identified using HMMER 3.1b1. The positions of the specific amino acids within these domains were computationally annotated. Canonical transcripts of each gene and KRAB domains were obtained from the ENSEMBL database. The *z*-scores for the ZNF domains and DNA fingerprint positions were calculated with the number of SNVs normalized to the genes number of ZNF domains, with *x* being the normalized number of SNVs within the zinc finger domains of each KZFP. The z-scores were calculated on the basis of the whole-genome sequencing cohort from gnomAD only to maximize the number of KZFPs included and avoid any coverage bias, as some KZFPs contain exons that are badly covered with exome sequencing.

$$Z-\mathrm{score}=\frac{\left(x-\mathrm{mean}(\mathrm{SNV}\;\mathrm{count})\right)}{\mathrm{sd}(\mathrm{SNV}\;\mathrm{count})}.$$

### Data availability

All raw and processed data have been submitted to the Gene Expression Omnibus database (accession no. GSE115387).

## Supplementary Material

Supplemental Material
