# Supplementary material for: ZNF445 is a primary regulator of genomic imprinting
Source: Genes Dev. 2019 Jan 1;33(1-2):49–54. doi: 10.1101/gad.320069.118 (PMC6317318; doi:10.1101/gad.320069.118)
Supplement: Supplemental Material [file supp_33_1-2_49__index.html]

ZNF445 is a primary regulator of genomic imprinting — Supplemental Material 

# ZNF445 is a primary regulator of genomic imprinting

## Supplemental Material

- Supplemental\_Materials.pdf
