# Supplementary material for: ZNF445 is a primary regulator of genomic imprinting
Source: Genes Dev. 2019 Jan 1;33(1-2):49–54. doi: 10.1101/gad.320069.118 (PMC6317318; doi:10.1101/gad.320069.118)
Supplement: Supplemental Material [file supp_33.1-2.49_Supplemental_Materials.pdf]

Supplemental_Methods

Immunoblotting

The procedure was previously described (Coluccio et al. 2018). Primary antibody used was anti-HA HRP (Sigma-Aldrich, #12013819001), anti-beta Actin HRP (ab20272, Abcam), anti-ZNF445 (PA5-52322, ThermoFisher).

RT-PCR using mouse tissues

Total RNA was extracted using TRIzol (Invitrogen), treated it with DNaseI (Thermo Scientific) and purified by ethanol precipitation. The cDNA was synthesized with random hexamer primer using RevertAid First Strand cDNA Synthesis Kit (Thermo Scientific). Gene expression was analyzed using LightCycler 480 (Roshe).

Phylogenetic anaylsis

Phylogenetic anaylsis of KZFPs evolution was performed and published in reference (Imbeault et al. 2017). Screenshot from genomic loci were taken from the ENSEMBL database. Alignments of the zinc finger prints and KRAB domains, and generation of the phylogenetic relations were performed with MAFFT (Kuraku et al. 2013) software and Phylo.io (Robinson et al. 2016), using default parameters. Colours were assigned using a conservation threshold >30%.

Bak M, Boonen SE, Dahl C, Hahnemann JMD, Mackay DJDG, Tümer Z, Grønskov K, Temple IK, Guldberg P, Tommerup N. 2016. Genome-wide DNA methylation analysis of transient neonatal diabetes type 1 patients with mutations in ZFP57. *BMC Med Genet* **17**: 29. http://bmcmedgenet.biomedcentral.com/articles/10.1186/s12881-016-0292-4.

Boonen SE, Mackay DJG, Hahnemann JMD, Docherty L, Gronskov K, Lehmann A,

Larsen LG, Haemers AP, Kockaerts Y, Dooms L, et al. 2013. Transient neonatal diabetes, ZFP57, and hypomethylation of multiple imprinted loci. *Diabetes Care* **36**: 505–512.

Coluccio A, Ecco G, Duc J, Offner S, Turelli P, Trono D. 2018. Individual retrotransposon integrants are differentially controlled by KZFP/KAP1-dependent histone methylation, DNA methylation and TET-mediated hydroxymethylation in naïve embryonic stem cells. *Epigenetics and Chromatin* **11**: 1–18.

Court F, Martin-Trujillo A, Romanelli V, Garin I, Iglesias-Platas I, Salafsky I, Guitart M, Perez de Nanclares G, Lapunzina P, Monk D. 2013. Genome-Wide Allelic Methylation Analysis Reveals Disease-Specific Susceptibility to Multiple Methylation Defects in Imprinting Syndromes. *Hum Mutat* **34**: 595–602.

Illingworth RS, Gruenewald-Schneider U, Webb S, Kerr ARW, James KD, Turner DJ, Smith C, Harrison DJ, Andrews R, Bird AP. 2010. Orphan CpG Islands Identify numerous conserved promoters in the mammalian genome. *PLoS Genet* **6**.

Imbeault M, Helleboid PY, Trono D. 2017. KRAB zinc-finger proteins contribute to the evolution of gene regulatory networks. *Nature* **543**: 550–554.

Kuraku S, Zmasek CM, Nishimura O, Katoh K. 2013. aLeaves facilitates on-demand exploration of metazoan gene family trees on MAFFT sequence alignment server with enhanced interactivity. *Nucleic Acids Res* **41**: W22–W28.

Okae H, Chiba H, Hiura H, Hamada H, Sato A, Utsunomiya T, Kikuchi H, Yoshida H,

Tanaka A, Suyama M, et al. 2014. Genome-Wide Analysis of DNA Methylation Dynamics during Early Human Development ed. R.J. Oakey. *PLoS Genet* **10**: e1004868.

Padmanabhan N, Jia D, Geary-Joo C, Wu X, Ferguson-Smith AC, Fung E, Bieda MC, Snyder FF, Gravel RA, Cross JC, et al. 2013. Mutation in folate metabolism causes epigenetic instability and transgenerational effects on development. *Cell* **155**: 81–93.

Riesewijk AM, Schepens MT, Welch TR, Van Den Berg-Loonen EM, Mariman EM, Ropers HH, Kalscheuer VM. 1996. Maternal-specific methylation of the human IGF2R gene is not accompanied by allele-specific transcription. *Genomics* **31**: 158–166.

Robinson O, Dylus D, Dessimoz C. 2016. Phylo.io: interactive viewing and comparison of large phylogenetic trees on the web.

Smits G, Mungall AJ, Griffiths-Jones S, Smith P, Beury D, Matthews L, Rogers J, Pask AJ, Shaw G, VandeBerg JL, et al. 2008. Conservation of the H19 noncoding RNA and H19-IGF2 imprinting mechanism in therians. *Nat Genet* **40**: 971–976.

Strogantsev R, Krueger F, Yamazawa K, Shi H, Gould P, Goldman-Roberts M, McEwen K, Sun B, Pedersen R, Ferguson-Smith AC. 2015. Allele-specific binding of ZFP57 in the epigenetic regulation of imprinted and non-imprinted monoallelic expression. *Genome Biol* **16**: 1–18. http://genomebiology.com/2015/16/1/112.

Sun B, Ito M, Mendjan S, Ito Y, Brons IGM, Murrell A, Vallier L, Ferguson-Smith AC, Pedersen RA. 2012. Status of genomic imprinting in epigenetically distinct pluripotent stem cells. *Stem Cells* **30**: 161–168.

Suzuki S, Ono R, Narita T, Pask AJ, Shaw G, Wang C, Kohda T, Alsop AE, Marshall Graves JA, Kohara Y, et al. 2007. Retrotransposon silencing by DNA methylation can drive mammalian genomic imprinting. *PLoS Genet* **3**.

Tomizawa S, Kobayashi H, Watanabe T, Andrews S, Hata K, Kelsey G, Sasaki H. 2011. Dynamic stage-specific changes in imprinted differentially methylated regions during early mammalian development and prevalence of non-CpG methylation in oocytes. *Development* **138**: 811–820. http://dev.biologists.org/cgi/doi/10.1242/dev.061416.

Woodfine K, Huddleston JE, Murrell A. 2011. Quantitative analysis of DNA methylation at all human imprinted regions reveals preservation of epigenetic stability in adult somatic tissue. *Epigenetics and Chromatin* **4**: 1–13.

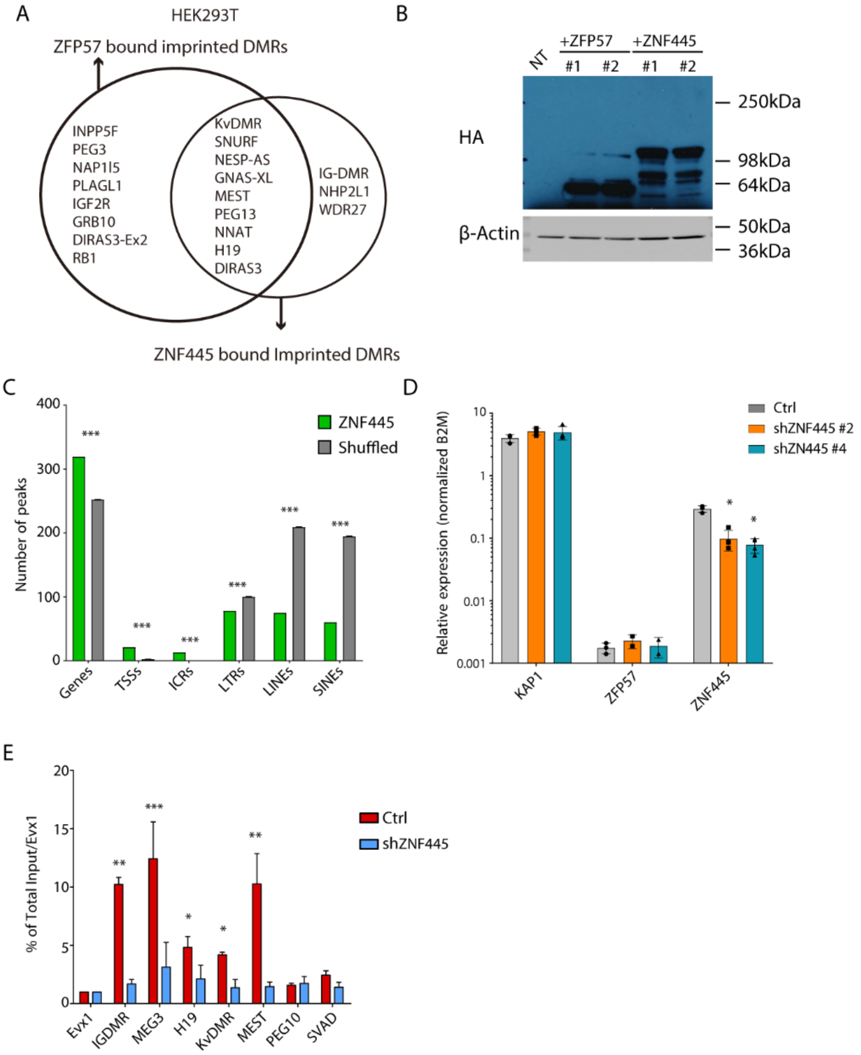

Fig. S1. Genomic binding of ZNF445 in human embryonic stem cells. (A) Venn-diagram of imprinted DMRs bound by ZFP57 and/or ZNF445 in HEK293T cells (Imbeault et al. 2017). (B) Western blot using anti HA and β-Actin antibodies HA-tagged ZFP57 or ZNF445 overexpressing hESCs. (C) Enrichment of ZNF445 (or shuffled peaks) on indicated genomic regions in hESCs. Statistical significance was calculated with Fisher's exact test, *p<0.05, **p<0.01, ***p<0.001. (D) Relative expression of indicated genes measured by RT-qPCR on *ZNF445* knockdown cells or control. Data are normalized to housekeeping gene *B2M*. The bars represent the mean±s.d. and single values are plotted for each replicate. *p<0.05, Student's t test. n=3. (E) ChIP-qPCR on hESCs wild-type or knockdown for ZNF445 using an antibody against endogenous ZNF445. The bars represent the mean+s.d. Student's t test. n=2. *p<0.05, **p<0.01, ***p<0.001.

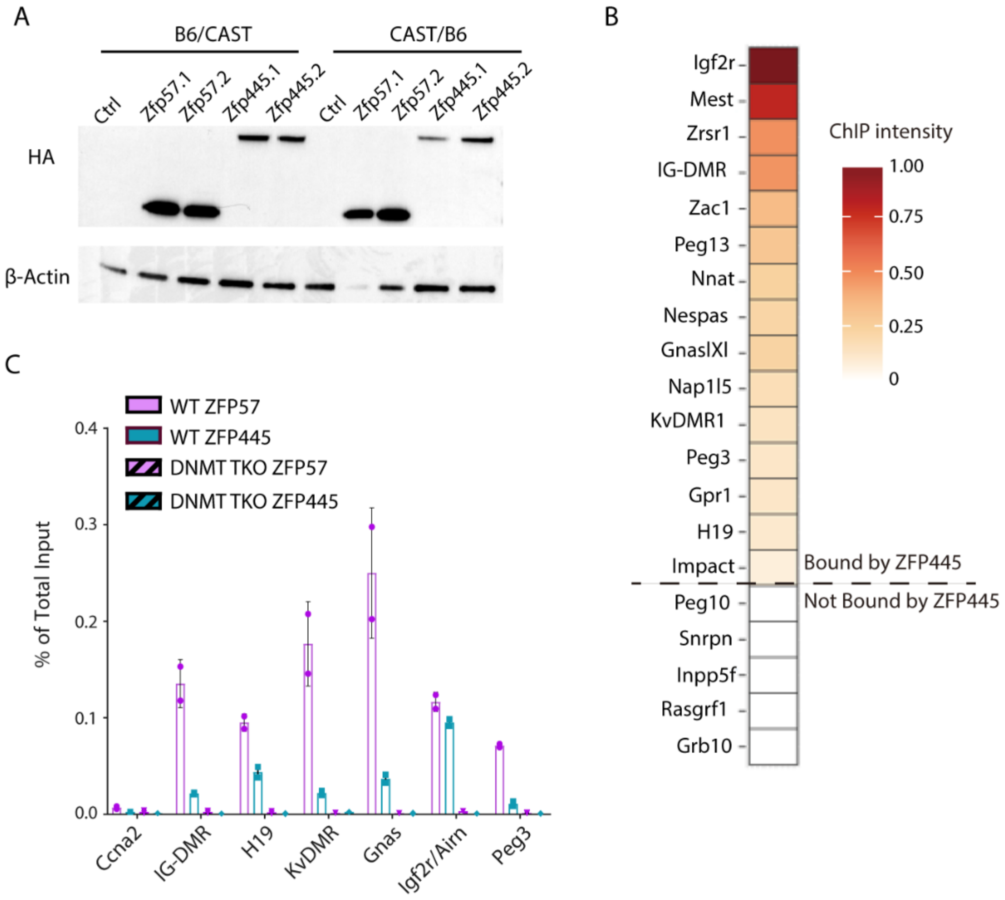

Fig. S2. Genomic binding of ZFP445 in murine embryonic stem cells. (A) Western Blot showing overexpression of HA-tagged forms of ZFP57 and ZFP445 in murine ES cells B6/CAST and CAST/B6. (B) ZFP445 ChIP intensity at ICRs in mES cells. (C) ChIP-qPCR on HA-tagged forms of ZFP57 and ZFP445 on ICRs in wild type and *Dnmts* triple knockout cells. The bars represent the mean±s.d. and single values are plotted for each replicate. n=2.

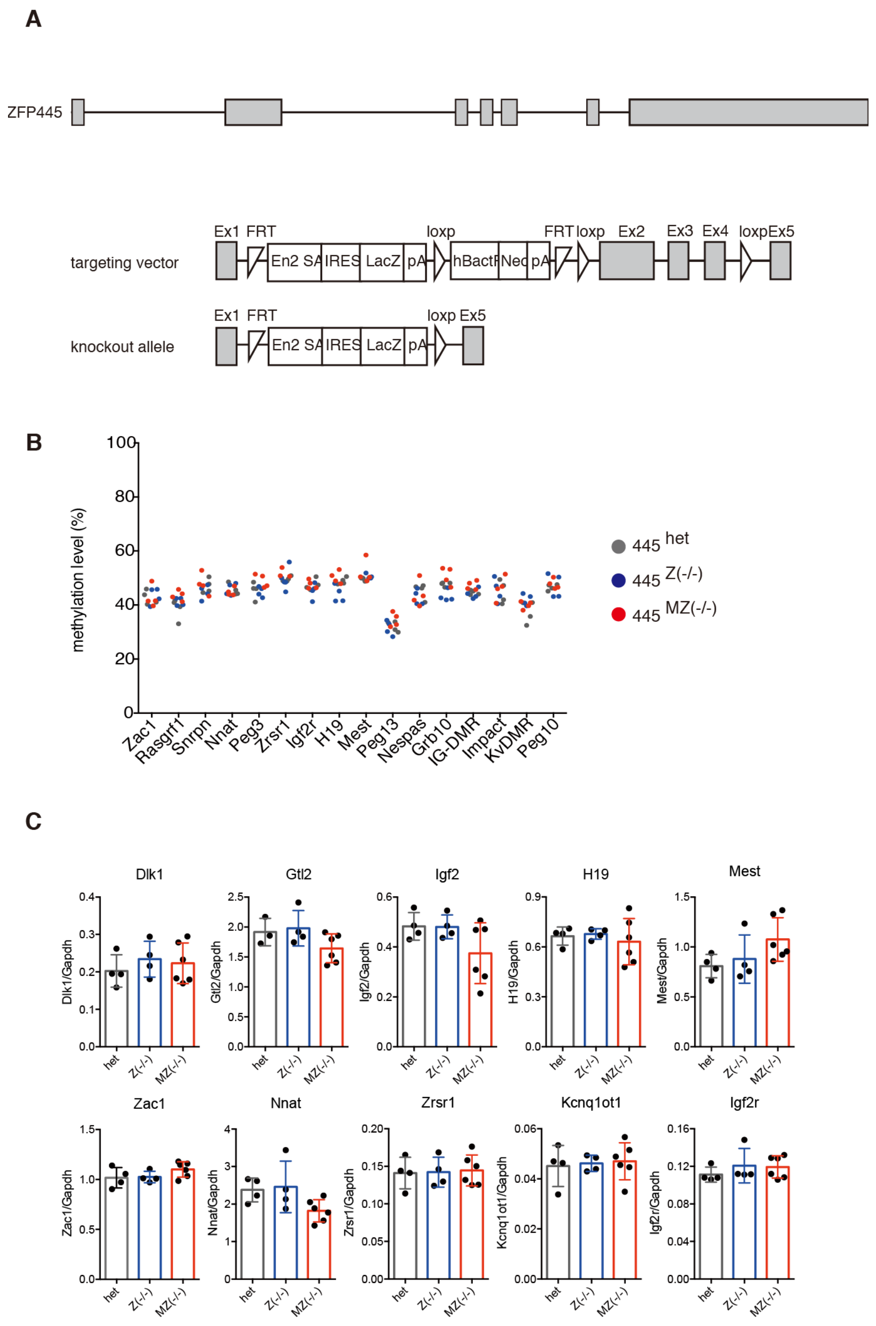

Fig. S3. Targeted deletion of murine ZFP445 alone does not influence the methylation state of ICRs and expression of imprinted genes. (A) Schematic representation of the genetic knockout strategy. (B) Methylation levels measured by pyrosequencing in liver at E12.5. Each dot represents the average methylation level of analysed CpG sites in each sample. (C) Expression of imprinted genes measured by RT-qPCR in brain at E12.5. Each dot represents individual sample.

A

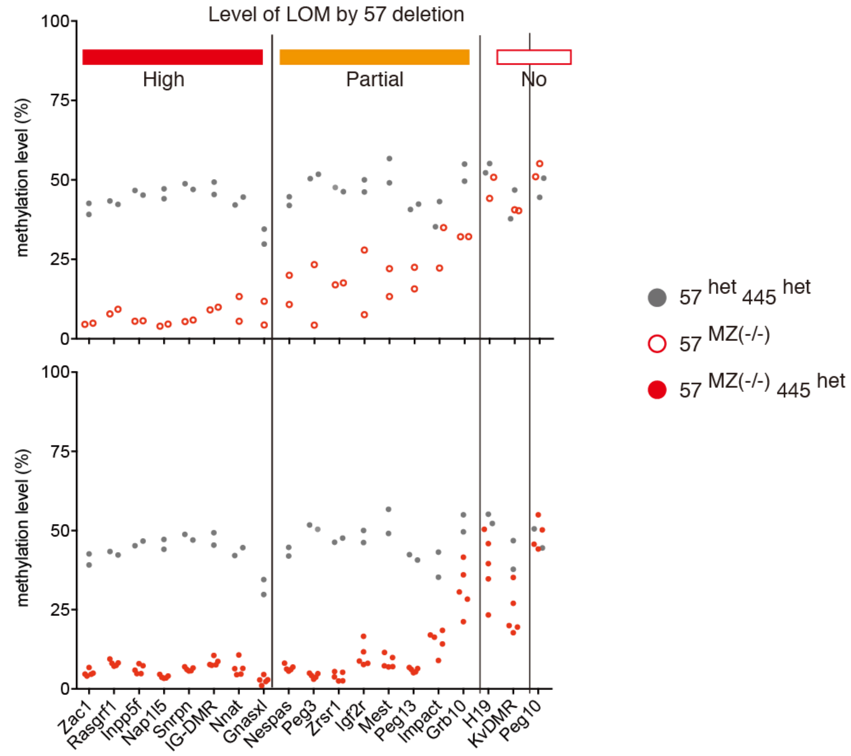

B

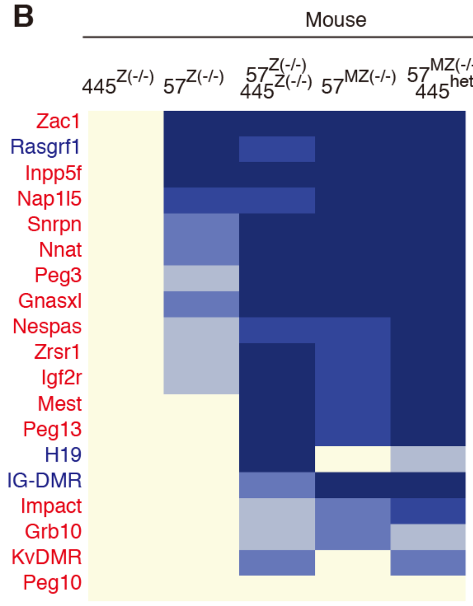

	Mouse		Human			No of ZFP57 binding motifs		
	57 binding	445 binding	57 binding	445 binding	LOM by mutation	mouse	human	Imprinted in Marsupials
Zac1	yes	yes	yes		yes	6	6	
Rasgrf1	yes					9		
Inpp5f	yes		yes		yes	6	4	
Nap1l5	yes	yes	yes		yes	5	6	
Snrpn	yes		yes	yes	no	10	3	
Nnat	yes	yes	yes	yes		6	5	
Peg3	yes	yes	yes		yes	8	11	
Gnasxl	yes	yes	yes	yes		7	5	
Nespas	yes	yes	yes	yes	yes	7	5	
Zrsr1	yes	yes				2		
Igf2r	yes	yes	yes			8	1	yes*
Mest	yes	yes	yes	yes	yes	12	8	yes*
Peg13	yes	yes	yes	yes		5	9	
H19	yes	yes	yes	yes	no	6	12	yes
IG-DMR	yes	yes		yes	no	10	1	
Impact	yes	yes				5		
Grb10	yes		yes		yes	4	8	
KvDMR	yes	yes	yes	yes	yes	3	6	
Peg10	yes					3	0	yes

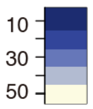

Fig. S4. Loss of methylation at imprinting control regions in double mutants for ZFP445 and ZFP57 (A) Methylation levels measured by pyrosequencing in embryonic brain of indicated genetic mutants at E11.5. Each dot represents average the methylation level of analysed CpG sites. *Zfp57*^{het}/*Zfp445*^{het} (n = 2) embryos were obtained from 2 litters by crossing female and male double heterozygotes, and *Zfp57*$^{MZ(-/-)}$ (n = 2) and *Zfp57*$^{MZ(-/-)}$/*Zfp445*^{het} (n = 5) embryos were obtained from 2 litters by crossing female *Zfp57*$^{Z(-/-)}$ with male *Zfp57*^{het}/*Zfp445*^{het}. **(**B) Summary of level of loss of methylation in the murine knockout models, methylation status in human patients with ZFP57 mutations (Bak et al. 2016; Court et al. 2013; Boonen et al. 2013), ZFP57 binding status in HEK293 cells (Imbeault et al. 2017), number of ZFP57 binding motifs and imprinting status in marsupials (Smits et al. 2008; Suzuki et al. 2007) for the indicated ICRs (the three paternal methylation imprints named in blue and the maternal methylation imprints in red). Methylation level of wild types is indicated in yellow and presented as 50% and average methylation levels of the indicated mutants was calculated and summarized as a heat map. *Differentially methylated regions are not identified in the corresponding eutherian locus. Published regions of murine ICRs and human imprinted DMRs were used (Okae et al. 2014; Riesewijk et al. 1996; Tomizawa et al. 2011; Illingworth et al. 2010) to determine the number of ZFP57 binding motifs.

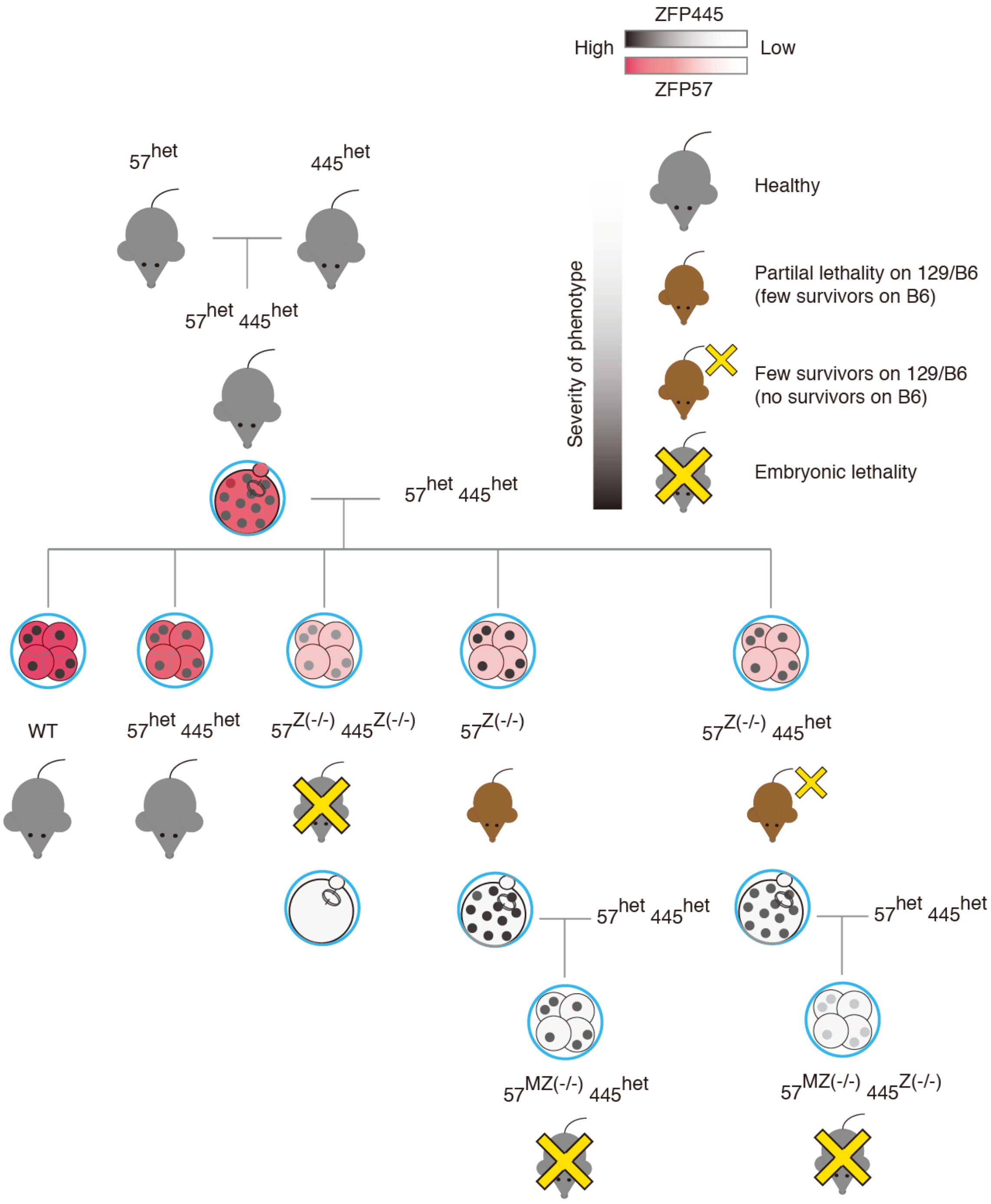

Fig. S5. Breeding strategy.

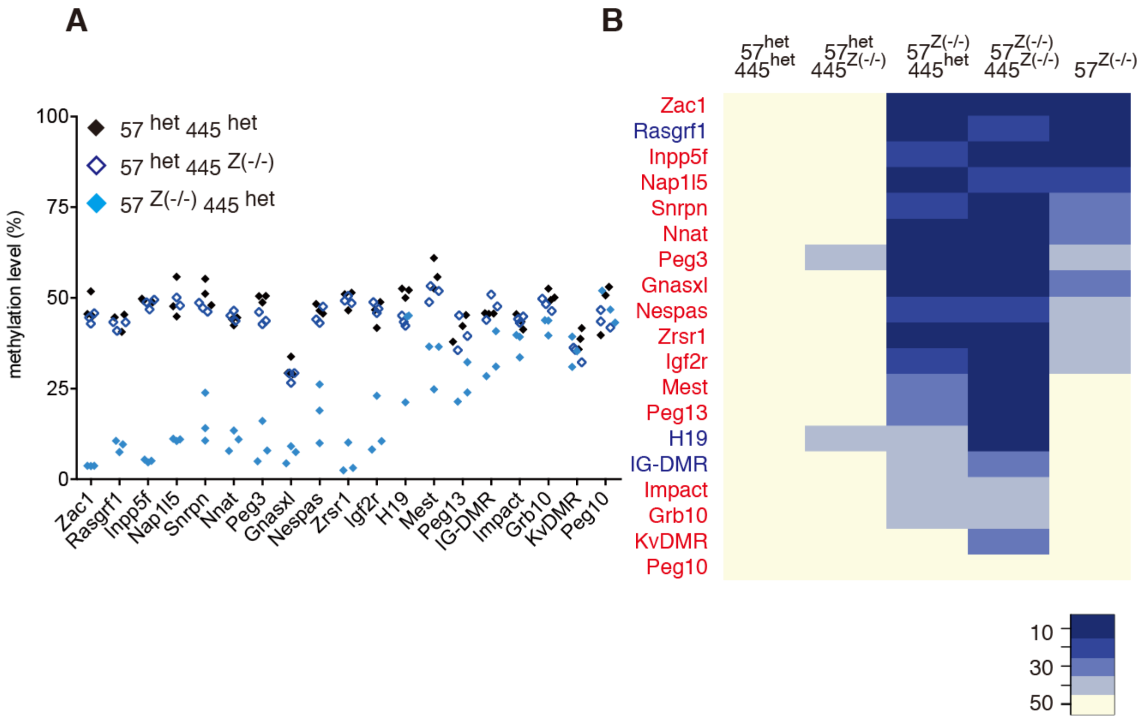

Fig. S6. Methylation levels at ICRs in animals double heterozygous for ZFP57 and ZFP445 and animals homozygous for one and heterozygous for the other KZFP. (A) Methylation levels were measured by pyrosequencing in embryonic brain of indicated genetic mutants at E11.5. (B). Heat map summary of methylation levels of wild type and indicated mutants with yellow representing normal imprinting. *Zfp57*$^{Z(-/-)}$/*Zfp445*^{het} mutants showed similar effects on methylation at ICRs as the *Zfp57*$^{Z(-/-)}$/*Zfp445*$^{Z(-/-)}$ mutants. In contrast there was little heterozygous effect of *Zfp57* when *Zfp445* was deleted indicating a primary role for ZFP57 and a more supplementary role for ZFP445 at most ICRs (n = 3, for each genotype).

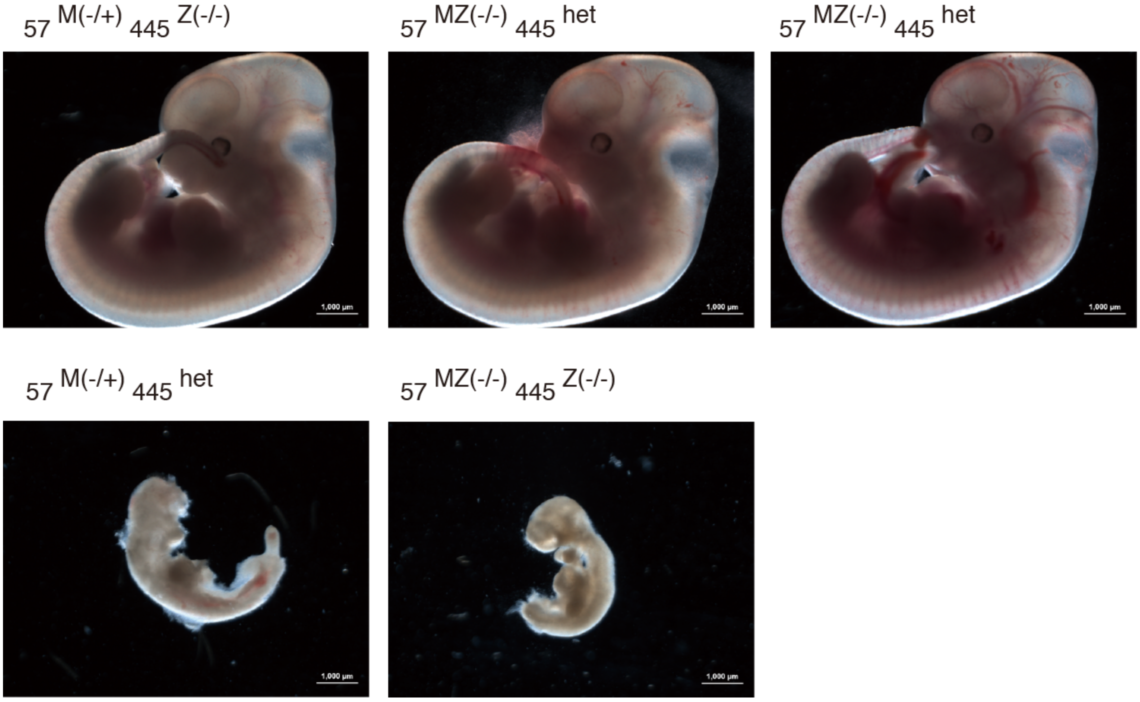

Fig. S7. Images of embryos derived from crossing female *Zfp57*$^{\mathbf{Z(-/-)}}$/*Zfp445*$^{\mathbf{het}}$ and male *Zfp57*$^{\mathbf{het}}$/*Zfp445*$^{\mathbf{Z(-/-)}}$ mutant mice. The female *Zfp57*$^{Z(-/-)}$/*Zfp445*^{het} mouse was sacrificed at day 11.5 of gestation. Scale bar = 1mm. DNA was isolated for methylation analysis from yolk sac and embryo of the *Zfp57*$^{MZ(-/-)}$/*Zfp445*$^{Z(-/-)}$ mutant and yolk sac of the *Zfp57*$^{M(-/+)}$/*Zfp445*^{het} mutant.

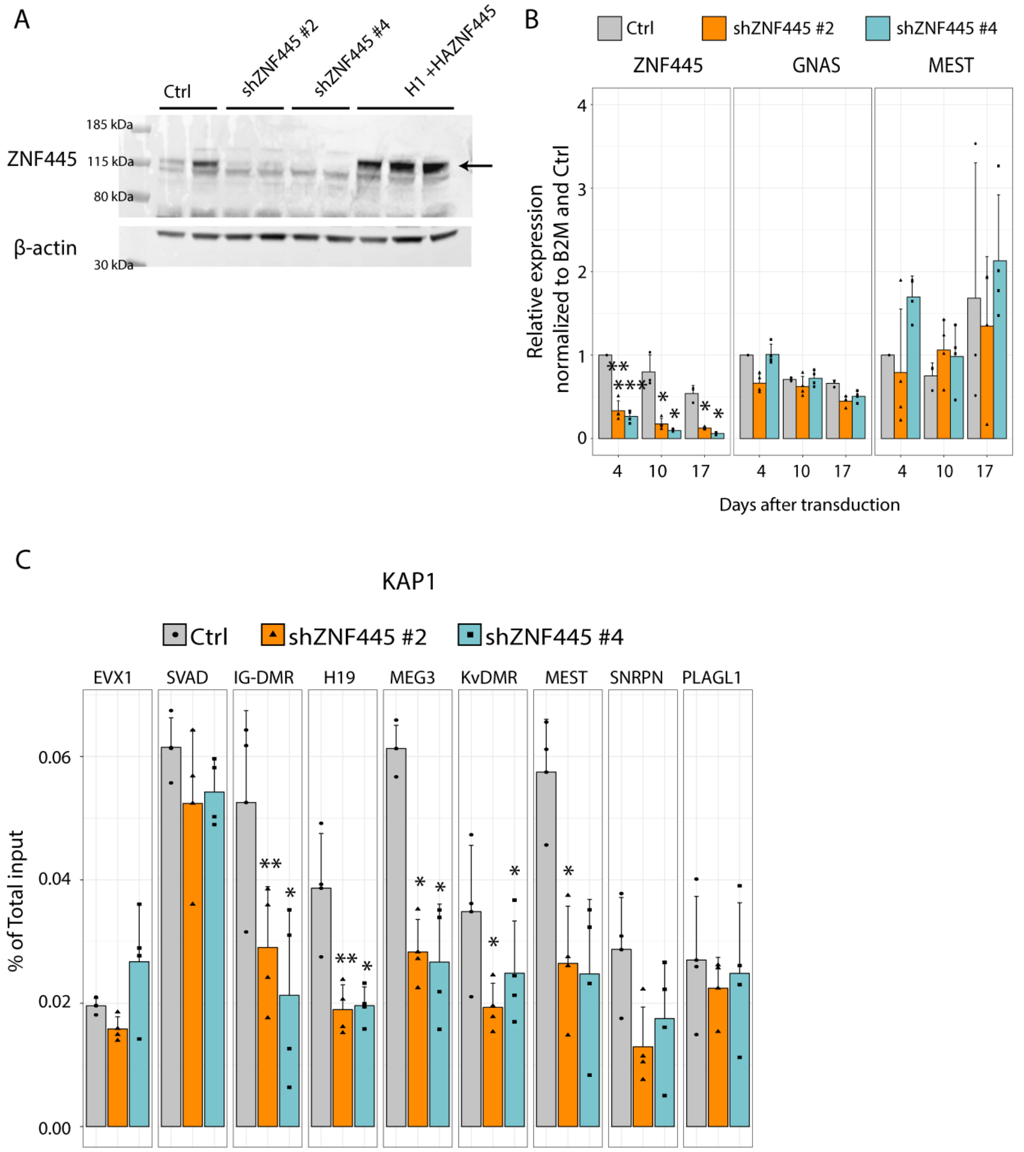

Fig. S8. Depletion of ZNF445 from human embryonic stem cells leads to loss of KAP1 recruitment at ICRs. A) Western blot for endogenous ZNF445 protein in hESCs either wild type, knockdown or overexpressing HA-ZNF445. Actinβ is used as a loading control. B) Relative expression of imprinted genes measured by RT-qPCR in ZNF445 knockdown cells or control. Data are normalized with the housekeeping gene *B2M* for ZNF445. The bars represent the mean+s.d. and single values are plotted for each replicate. *p<0.05, Student's t test, n=4 C) KAP1 enrichment at indicated genomic loci found by ChIP-qPCR in wild-type and *ZNF445* knockdown hESCs (using two different shRNAs). The bars represent the mean+s.d. and single values are plotted for each replicate. *p<0.05, **p<0.01, Student's t test, n=4.

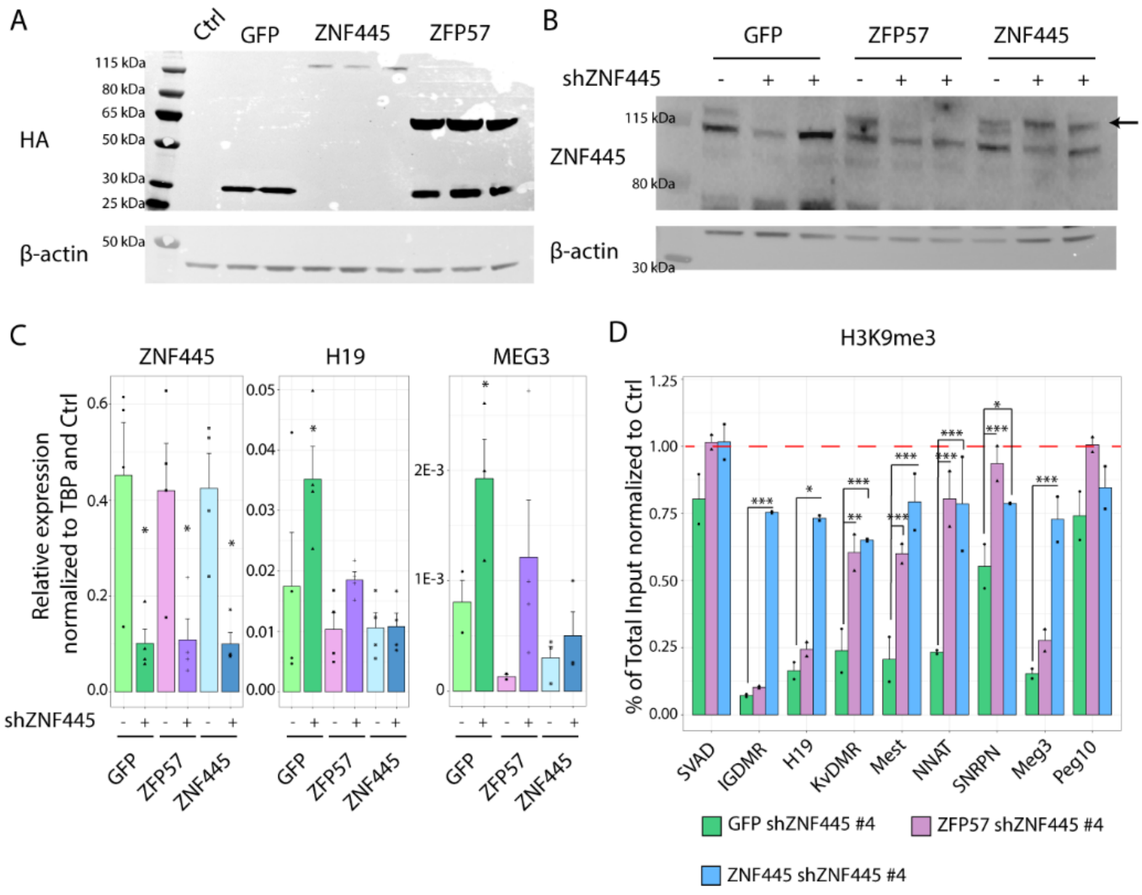

Fig. S9. Complementation of ZNF445 depletion rescues imprinted gene regulation and H3K9me3 enrichment at ICRs. (A,B) Western blot showing expression of HA-tagged GFP, ZFP57 or ZNF445 (A) and the levels of endogenous ZNF445 upon knockdown and/or complementation (B). (C) Relative expression of imprinted genes measured by RT-qPCR. Primers for ZNF445 are specific for the endogenous protein and do not amplify the codon-optimized overexpressed HA-ZNF445. Data are normalized with housekeeping gene *TBP*. The bars represent the mean+s.d. and single values are plotted for each replicate. *p<0.05, **p<0.01, ***p<0.001, Student's t test. n=4. (D) Enrichment of H3K9me3 normalized to control cells without ZNF445 knockdown for each sample. The bars represent the mean+s.d. and single values are plotted for each replicate. *p<0.05, **p<0.01, ***p<0.001 Student's t test. n=2.

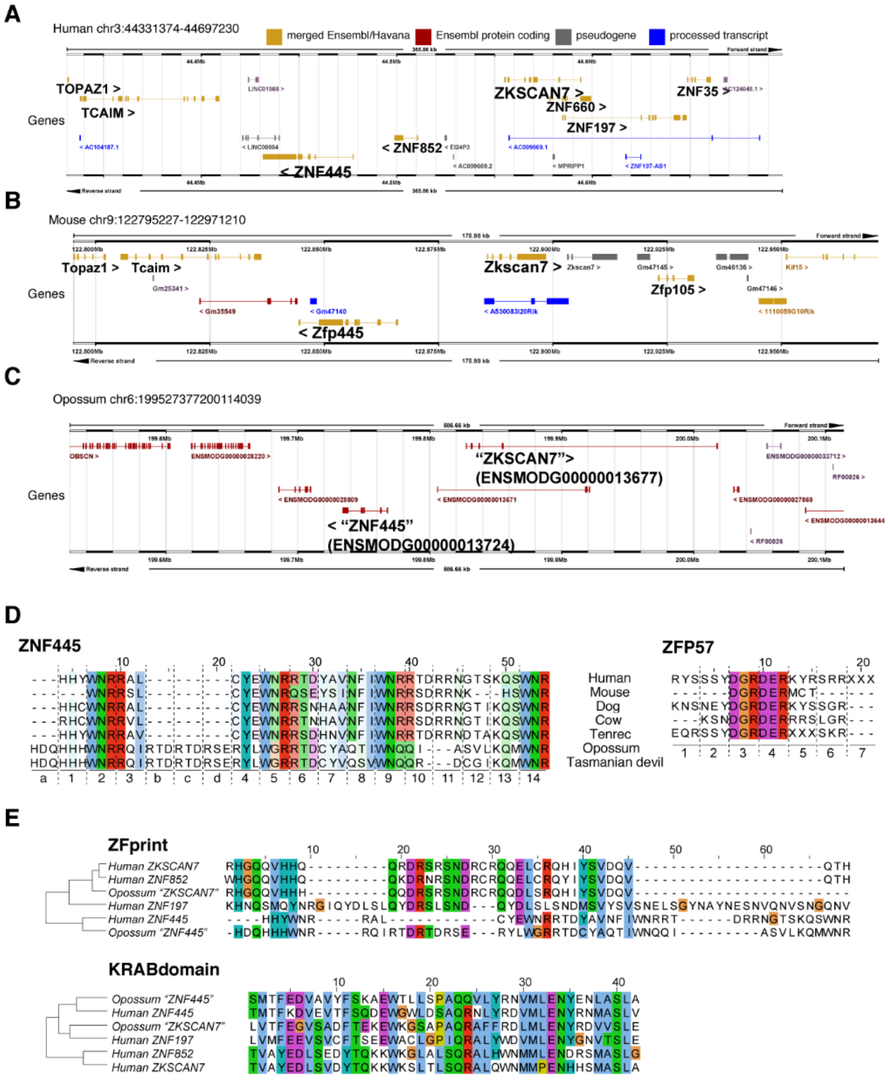

Fig. S10. Phylogenetic analysis of ZNF445 evolution in therian genomes. (A,B,C) Genomic location of human ZNF445 (A), murine ZFP445 (B) and the Opossum putative orthologue (C) with a schematic representation of the various domains of the ZNF445 protein. SCAN: SRE-ZBP, CTfin51, AW-1 and Number 18 cDNA. KRAB: Krüppel associated box. ZF: zinc finger. (E) Alignment between the 3 amino acids of each zinc-finger motif corresponding to the zinc-finger signatures of ZNF445 and ZFP57 across several species corresponding to Fig. 4A. Alignment is shown with Jalview software and coloured according to ClustalX color scheme with conservation threshold >30%. F) Alignment of the zinc-finger signature and the KRAB domains of the KZFPs present in the ZNF445 cluster in human and opossum.

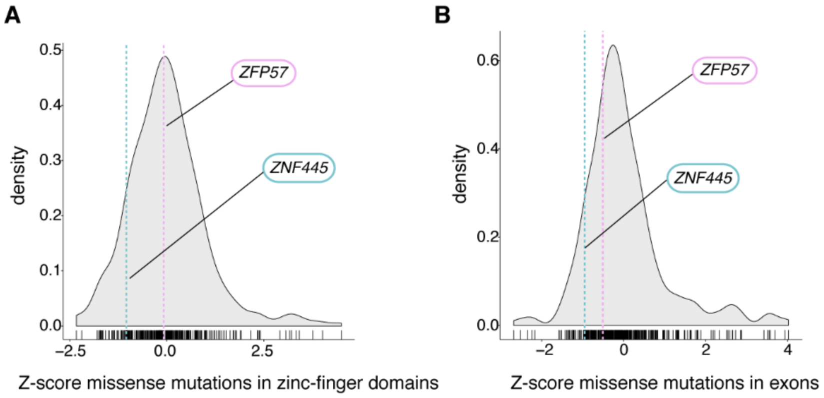

Fig. S11. ZNF445 has a low occurrence of missense mutation across the human population. Z-scores for the occurrence of missense variants in the zinc finger domain (A) or in any exon (B) for all the KZFPs.

Table S1 ZFP57 and ZNF445 binding at imprinted DMRs in hESCs and HEK293 cells

Name	position	Methylated allele	Binding in hES			Binding in HEK		No. of 57 binding motifs
			KAP1	445	57	445	57	
**DMRs commonly imprinted in human and mouse**								
INPP5F	chr10: 121578046-121578727	Maternal			Yes		Yes	4
KvDMR1	chr11: 2719948-2722259		Yes	Yes	Yes	Yes	Yes	6
SNURF	chr15: 25200004-25201976				Yes	Yes	Yes	3
PEG3	chr19: 57348493-57353271				Yes		Yes	11
NESP-AS	chr20: 57425649-57428033				Yes	Yes	Yes	5
GNAS-XL	chr20: 57428905-57431463			Yes	Yes	Yes	Yes	5
NAP1l5	chr4: 89618184-89619237				Yes		Yes	6
PLAGL1	chr6: 144328078-144329888		Yes		Yes		Yes	6
IGF2R	chr6: 160426375-160427561		Yes		Yes		Yes	1
GRB10	chr7: 50848726-50851312				Yes		Yes	8
MEST	chr7: 130130122-130134388		Yes	Yes	Yes	Yes	Yes	8
TRAPPC 9/PEG13	chr8: 141108147-141111081			Yes	Yes	Yes	Yes	9
BLCAP/N NAT	chr20: 36148604-36150528			Yes	Yes	Yes	Yes	5
MCTS2	chr20: 30134663-30135933							0
PEG10	chr7: 94285537-94287960		Yes					0
H19	chr11: 2018812-2024740	Paternal	Yes	Yes	Yes	Yes	Yes	12
IG-DMR	chr14: 101275427-101278058		Yes	Yes		Yes		1
**Imprinted DMRs in human (not imprinted in mouse)**								
DIRAS3	chr1: 68515433-68517545	Maternal		Yes	Yes	Yes	Yes	3
DIRAS3-Ex2	chr1: 68512505-68513486						Yes	1
RB1	chr13: 48892341-48895763				Yes		Yes	1
NHP2L1	chr22: 42077774-42078873					Yes		2
WDR27	chr6: 170054504-170055618		Yes			Yes		1
HTR5A	chr7: 154862719-154863382				Yes			2
PPIEL	chr1: 40024626-40025540							0
HTR5A	chr7: 154862719-154863382							2
IGF1R	chr15: 99408496-99409650							0
ZNF597	chr16: 3481801-3482388							1
ZNF331	chr19: 54040510-54042212							0
ZNF331	chr19: 54057086-54058425							0
L3MBTL	chr20: 42142365-42144040							2
FAM50B	chr6: 3849082-3850359							3
CXORF5 6	chr8: 37604992-37606088							0

Regions of imprinted DMRs were identified in the published papers (Okae et al. 2014; Riesewijk et al. 1996)

Table S2 Divergence from Mendelian genetic ratios in heterozygous/homozygous intercrosses between ZFP57 and ZFP445 and the influence of genetic background on outcomes.

(A) Survival rates of *zfp57* Z(-/-) and *zfp445* Z(-/-) mutants at P10 on C57BL/6

Targeted Gene	Parental genotypes		No. of litters	Genotype of pups (%)		
	Female	Male		WT	Het	Z(-/-)
Zfp57	Het	Het	94	133 (36)	234 (63.4)	2 (0.5)
Zfp445	Het	Z(-/-)	14	-	66 (84.6)	12 (15.4)

(B) Genotype of E11.5 embryos from the cross between female and male double heterozygous mice on C57BL/6

**Genotype of embryos**	**No. of embryos at E11.5**	**Observed Ratio (%)**	**Expected ratio (%)**
**WT**	3	5.5	6.25
**57 ^{het} 445 ^{het}**	14	25.5	25
**445 ^{het}**	7	12.7	12.5
**57 ^{het}**	9	16.4	12.5
**445 $^{Z(-/-)}$**	3	5.5	6.25
**57 $^{Z(-/-)}$**	2	3.6	6.25
**57 ^{het} 445 $^{Z(-/-)}$**	5	9.1	12.5
**57 $^{Z(-/-)}$ 445 ^{het}**	7	12.7	12.5
**57 $^{Z(-/-)}$ 445 $^{Z(-/-)}$**	5	9.1	6.25

(C) Genotype of pups from the cross between female and male double heterozygous mice on 129/B6 mixed background (24 litters)

**Genotype of pups**	**No. of pups at P10**	**Observed Ratio (%)**	**Expected ratio (%)**
**WT**	12	8.5	6.25
**57 ^{het} 445 ^{het}**	56	38.4	25
**445 ^{het}**	31	21.2	12.5
**57 ^{het}**	27	18.5	12.5
**445 $^{Z(-/-)}$**	7	4.8	6.25
**57 $^{Z(-/-)}$**	3	2.1	6.25
**57 ^{het} 445 $^{Z(-/-)}$**	8	5.5	12.5
***57 $^{Z(-/-)}$ 445 ^{het}**	2	1.4	12.5
**57 $^{Z(-/-)}$ 445 $^{Z(-/-)}$**	0	0	6.25

(D) Genotype of pups from the cross between female double heterozygous and male *Zfp57$^{Z(-/-)}$/Zfp445 ^{het}* mice on mixed 129/B6 background (11 litters)

**Genotype of pups**	**No. of pups at P10**	**Observed Ratio (%)**	**Expected ratio (%)**
**57 ^{het} 445 ^{het}**	30	46.2	25
**57 ^{het}**	17	26.2	12.5
**57 $^{Z(-/-)}$**	11	16.9	12.5
**57 ^{het} 445 $^{Z(-/-)}$**	7	10.8	12.5
**57 $^{Z(-/-)}$ 445 ^{het}**	0	0	25
**57 $^{Z(-/-)}$ 445 $^{Z(-/-)}$**	0	0	12.5

(E) Genotype of pups from the cross between female double heterozygous and male *Zfp57*^{het}/*Zfp445*$^{Z(-/-)}$ mice on mixed 129/B6 background (13 litters)

Genotype of pups	No. of pups at P10	Observed Ratio (%)	Expected ratio (%)
**57 ^{het} 445 ^{het}**	29	42	25
**445 ^{het}**	14	20.3	12.5
**445 $^{Z(-/-)}$**	13	18.8	12.5
**57 ^{het} 445 $^{Z(-/-)}$**	12	17.4	25
***57 $^{Z(-/-)}$ 445 ^{het}**	1	1.4	12.5
**57 $^{Z(-/-)}$ 445 $^{Z(-/-)}$**	0	0	12.5

(F) Genotype of pups from the cross between female ZFP57 heterozygous and male *Zfp57*^{het}/*Zfp445*$^{Z(-/-)}$ mice on mixed 129/B6 background (68 litters)

Genotype of pups	No. of pups at P10	Observed Ratio (%)	Expected ratio (%)
**57 ^{het} 445 ^{het}**	246	52.8	50
**445 ^{het}**	146	31.3	25
***57 $^{Z(-/-)}$ 445 ^{het}**	6	1.3	25

(G) Genotype of pups from the cross between female ZFP57 heterozygous and male *Zfp57*$^{(-/-)}$/*Zfp445*^{het} mice on mixed 129/B6 background (18 litters)

Genotype of pups	No. of pups at P10	Observed Ratio (%)	Expected ratio (%)
**57 ^{het} 445 ^{het}**	36	31.9	25
**57 ^{het}**	55	48.7	25
**57 $^{Z(-/-)}$**	20	17.7	25
***57 $^{Z(-/-)}$ 445 ^{het}**	2	1.8	25

*Only two *Zfp57* $^{Z(-/-)}$/*Zfp445*^{het} mutant mice were female.

(H) Genotype of E10.5 embryos from the cross between female *Zfp57*$^{(-/-)}$/*Zfp445*^{het} and male *Zfp57*^{het}/*Zfp445*$^{Z(-/-)}$ mice on mixed 129/B6 background (1 litter)

Genotype of embryos	No. of embryos	Observed Ratio (%)	Expected ratio (%)
**57 $^{M(-/+)}$ 445 ^{het}**	2	22.2	25
**57 $^{M(-/+)}$ 445 $^{Z(-/-)}$**	3	33.3	25
**57 $^{MZ(-/-)}$ 445 ^{het}**	4	44.4	25
**57 $^{MZ(-/-)}$ 445 $^{Z(-/-)}$**	0	0	25

Table S3

(A) List of primers used for methylation analysis in mouse and human

Mouse

DMRs	Forward	Reverse	Sequence	References
Zac1	TGTTAGGAGAGTGAGGTTGGAGAA	CATACAACCATCCCCTTAACT	GTTTTAGTTTAATTGAGTGATAAAT	
Rasgrf1	GGGAAGATTATTAGTTGGGGAGGTG	CAACAAAAACCAAAATATCAATCCTAAC	ATTAGAGTTAAATATAAAGAATGG	(Padmanabhan et al. 2013)
Inpp5f	AGGTGAGGTGTAGATAGAGGATG	ATACAACCCCACTAACACTTT	GGTGTAGATAGAGGATGT	
Nap1l5	GATTTTGGAGAGTAGGGGTTTGTAGAT	AAAACTTTATAAAAAACTTACCCAATT	GGAATTTTTTGTTAAATTTGGTT	
Snrpn	TTGGTAGTTGTTTTTTGGTAGGAT	TCCACAAACCCAACTAACCTTC	GTGTAGTTATTGTTTGGGA	(Sun et al. 2012)
IG-DMR	GTGGTTTGTTATGGGTAAGTTT	CCCTTCCCTCACTCCAAAAATTAA	TGGTTTATTGTATATAATGT	(Sun et al. 2012)
Nnat	AAAGGTATATATTTTGTTTTTAGAGAGAT	ACACACCCAAACCTACAAATT	CCCAAACCTACAAATTC	
Gnasxl	TGGTTTTTTAGGGGTTGAGGGA	AACCACCCACTACTTCCAATAACTT	GTGGTTTAGGGGTAGGTTA	
Nespas	GGGATGGTTTATGGGGGTTT	ATCTCAACCACTAACCCACTCC	GTGGTTTTTTAGGTTTGG	
Peg3	TTGGATTGGTTAGAGAGGAAGT	ACAATCTAATACACCCACACTAA	GGAGAGATGTTTATTTTG	(Sun et al. 2012)
Zrsr1	ATGGTTAGGTTGAGAGTTTTGGAAGTTT	TCCCTCAACAACCACTCTTCATA	TTTTGGAAGTTTTATTAGAGG	
Igf2r	GGGTGAAGATTTTTGGGTTATAAG	CCCCCCCCAATACAACAA	TTTATTGTTTATTAGTGTTTTGAAT	
Mest	AAGTGGGTGTAGTAATAAGAATTTTAGT	TATTAACCCCCTACCCCCTCTTTCCT	TTGGGGAGGGATTTT	
Peg13	TTGGATGAGTTATTATATAAGGTTTAAAA	ACTAAACCAACCCCTTTACTACAACTCTAT	AAATTTTAATAAGATGGGTTAAT	
Grb10	ATTTTTTGGAAGTTGAGAAGAG	ACAACCTCCCCAATAACCATCCC	AACCCCCCTCCACCT	(Padmanabhan et al. 2013)
Impact	GGGTTGATTGGTGTGTAAGA	AAAACCCCTAAACAACCTACTTAATACA	TGTGTGTGTTTGGGTATA	
H19	GGGGGGTAGGATATATGTATTTTT	ACCTCATAAAACCCATAACTATAAAATCAT	GTGTGTAAAGATTAGGG	(Sun et al. 2012)
KvDMR	AGAAGGGTGTTGAAGAAAAATT	ATCCTAAACCTAAACCTCCATAA	GTTGAGAAGTTAAGTGGA	(Sun et al. 2012)
Peg10	AATTTTGTTAAGTTTTTAGTGGTTAGAT	CACTTAAAAATACAAAACCAATCACTT	CACAATTCCATCAATAACT	(Padmanabhan et al. 2013)

Human

IG-DMR	TTTTATTATTGAATTGGGTTTGTTAGT	ACAATTCCTACTACAAAATTTCAACA	GGGTTTGTTAGTAGTT	
H19	TATGGGTATTTTTGGAGGTTTTTT	AAATCCCAAACCATAACACTAAAAC	TTGGTTGTAGTTGTGGA	
MEG3	TTGTGTTTGAATTTATTTTGTTT	CCCAAATTCTATAACAAATTACT	TTTGAATTTATTTTGTTTGG	
KvDMR	AGGGAAGTTTTAGGGTGTGAATTTTTAGAG	CCAAACCACCCACCTAACAAAAAAC	TGGTAATGTTTGGTATTT	(Woodfine et al. 2011)
MEST	AAGGGGGTTTTGTTTTTTTAATTGTG	AAACTCTATTAAACCCACCACCAAACTAAT	TTGTTGTAAAGGAAATTT	
SNRPN	TGGTTTTTTAGAATAAAGGATTTTAGGGTTTA	TAAAAATCCAATAACCCCCTCCCCC	TTAGGTTGTTTTTTGAGA	
PLAGL1	GGTTGAATGATAAATGGTAGATGT	ACCTTAACTTTACCCCCAC	TGGTAGGAGGAGGTTT	(Woodfine et al. 2011)

(B) List of primers used for RT-qPCR

Mouse

Genes	Forward	Reverse	Reference
Gapdh	AAGGGCTCATGACCACAGTC	GGATGCAGGGATGATGTTCT	(Strogantsev et al. 2015)
Dlk1	GGAGCTGGCGGTCAATAT	AACGCTGCTTAGATCTCCTCATCA	(Strogantsev et al. 2015)
Gtl2	GCTTCTCGAGGCCTGTCTAC	TTCGATGGAGAAGAGCGAGT	
H19	GCAATGCTGCCCCAGTAC	GACTAGGCGAGGGGAAGGC	
Igf2	CGCTTCAGTTTGTCTGTTCG	GGGGTGGCACAGTATGTCTC	
Zrsr1	ACTGGAGATAGAGCGGCAAA	CTAGCGGCCTCTTCCTTTTT	
Zac1	TTCGTCACCCTGGAGAAGTT	GGTCTGGAGGTGGTTCTTCA	
Nnat	AGAACTGCTCATCATCGGCT	TTCGAAAAGCGAATCCTACC	
Kcnq1ot1	AACGGAGCCCCTCACTCTCA	CTGGAGACCCCTGAGCTTTGTA	
Mest	CTGCTCTGCACTCATGGAAG	GGAAAGCCATGTAAAAGCACA	
Igf2r	CAGGCCGTCGACTTGGAC	ACCCACATTTCCACAGACGT	(Strogantsev et al. 2015)

Human

Genes	Forward	Reverse
ZNF445	AGCTCCAGGAGACCATGACT	GAATGGTCCCACCAGGGAAG
KCNQ1OT	GGGACACAGGAGTGTAAGCC	TGGTCTGGTGGGCTTTTGTT
MEG3	TGCATCAGGTAGGGGCTTTG	GTCAGGAAGCAGTGGGTTGA
H19	CAGGAGTGATGACGGGTGGAG	TCGCCCTGTCTGCACGATG
B2Micro	TGCTCGCGCTACTCTCTCTTT	TCTGCTGGATGACGTGAGTAAAC
TBP	GCC CGA AAC GCC GAA TAT A	CGT GGC TCT CTT ATC CTC ATG A
KAP1	AAG GAC ACT GTG CGC TCT AC	ACG TTG CAA TAG ACA GTA CGT TCA C
MEST	GGAAGTCTTCAGACTCTGTGGG	AAGGGCAATCACCCGATGAA
GNAS	GAGAAGCAGCTGCAGAAGGA	CCAGATTCTCCAGCACCCAG

(C) List of primers used for ChIP-qPCR and shRNAs sequence

**Mouse**	
**ChIP-qPCR**	
Ccna2 F	AGT AGC CCG CGA CTA TTG AAA T
Ccna2 R	GCG ACC GGC GCT TCT
IG-DMR F	TGTACACAATGCTGCCGTTC
IG-DMR R	CTCGCTAGTTCACGGAGGTC
H19 F	GCA CAG CGT GGA GAG TGA AC
H19 R	CAT TTC TTG GGT AGC TCC TTC AG
KvDMR F	AAACGAATACGGAGCCACTG
KvDMR R	GCGGGTTTCTTCTCTGAGTC
Gnas F	TGC CCA GGA ATA ATC TGC AGA
Gnas R	ATA CAG TCA CAT TGC CCG GT
Peg3 F	GCC ACT GCG GCA AAA CA
Peg3 R	GGT CTT CGC AAT CTA GCC ATC T
Airn_F	GGATTCGGAGGGTTTAGAGG
Airn_R	CAACTCAGCACAACCAAGGA
**Human**	
shZNF445 #2	CCGGATCAAACTTTACTCGTCATATCTCGAGATATGACGAGTAAAGTTTG ATTTTTTTG
shZNF445 #4	CCG GGC GCT ATA AAT GTA ATC TAT GCT CGA GCA TAG ATT ACA TTT ATA GCG CTT TTT G
**ChIP-qPCR**	
EVX1_F	CTGGGTGTCTCCCTCTCTCA
EVX1_R	AAAGGAAACCCGCAGCTAAT
SVAD_F	CTCGTTCACTCAGTGCTCAATG
SVAD_R	CTGGGAGGTGGAGGTTGTAG
H19_F	AAT TTG CTG TGC TCA TCA CG
H19_R	TCT TCG TAT CGG GCC ATA TC
KvDMR_F	ACA CAG CTC ACC TCA GCA AC
KvDMR_R	TCT CTC TGG GAG GGT TTG AA
SNRPN_F	TTC TAG AGG CCC CCT CTC AT
SNRPN_R	CCT CAC CGG AAT GAC CTG

IGDMR_F	CTG GCT TGA TCT TCC CTG AG
IGDMR_R	AGC TAG CAG TCT TGG GGT GA
MEST_F	GCG AAA ACT CTA CCG ACA GG
MEST_R	AAA TCT CAC CAC GAC GAT CC
PLAGL1_F	ATGAGAAACGCGACAGATGC
PLAGL1_R	AAAGTGCTTAGGACAGTGCC
MEG3_F	GAACCCCGGATTTCCTGTAT
MEG3_R	ACCCTTCTATTCGGGTGCTT
